# The HIV-1 Rev/RRE system is required for HIV-1 5' UTR *cis *elements to augment encapsidation of heterologous RNA into HIV-1 viral particles

**DOI:** 10.1186/1742-4690-8-51

**Published:** 2011-06-24

**Authors:** Adam S Cockrell, Henriette van Praag, Nicholas Santistevan, Hong Ma, Tal Kafri

**Affiliations:** 1 Gene Therapy Center University of North Carolina School of Medicine, Chapel Hill, North Carolina, USA; 2 Neuroplasticity and Behavior Unit, National Institute on Aging (NIA), Baltimore, Maryland, USA

## Abstract

**Background:**

The process of HIV-1 genomic RNA (gRNA) encapsidation is governed by a number of viral encoded components, most notably the Gag protein and gRNA *cis *elements in the canonical packaging signal (ψ). Also implicated in encapsidation are *cis *determinants in the R, U5, and PBS (primer binding site) from the 5' untranslated region (UTR). Although conventionally associated with nuclear export of HIV-1 RNA, there is a burgeoning role for the Rev/RRE in the encapsidation process. Pleiotropic effects exhibited by these *cis *and *trans *viral components may confound the ability to examine their independent, and combined, impact on encapsidation of RNA into HIV-1 viral particles in their innate viral context. We systematically reconstructed the HIV-1 packaging system in the context of a heterologous murine leukemia virus (MLV) vector RNA to elucidate a mechanism in which the Rev/RRE system is central to achieving efficient and specific encapsidation into HIV-1 viral particles.

**Results:**

We show for the first time that the Rev/RRE system can augment RNA encapsidation independent of all *cis *elements from the 5' UTR (R, U5, PBS, and ψ). Incorporation of all the 5' UTR *cis *elements did not enhance RNA encapsidation in the absence of the Rev/RRE system. In fact, we demonstrate that the Rev/RRE system is required for specific and efficient encapsidation commonly associated with the canonical packaging signal. The mechanism of Rev/RRE-mediated encapsidation is not a general phenomenon, since the combination of the Rev/RRE system and 5' UTR *cis *elements did not enhance encapsidation into MLV-derived viral particles. Lastly, we show that heterologous MLV RNAs conform to transduction properties commonly associated with HIV-1 viral particles, including *in vivo *transduction of non-dividing cells (i.e. mouse neurons); however, the cDNA forms are episomes predominantly in the 1-LTR circle form.

**Conclusions:**

Premised on encapsidation of a heterologous RNA into HIV-1 viral particles, our findings define a functional HIV-1 packaging system as comprising the 5' UTR *cis *elements, Gag, and the Rev/RRE system, in which the Rev/RRE system is required to make the RNA amenable to the ensuing interaction between Gag and the canonical packaging signal for subsequent encapsidation.

## Background

Specific and efficient encapsidation of HIV-1 gRNA into viral particles is a multifaceted process of relocating the gRNA following transcription in the nucleus to sites of particle assembly at the plasma membrane. *Cis *packaging signals in the viral RNA confer specific selection among the milieu of host cell RNAs through interactions with *trans *factors encoded by the virus, and host cell. The conventional canonical *cis *packaging signal (ψ) is a ~120 bp fragment comprised of four stem-loop structures located in the HIV-1 5' untranslated region (UTR), and extending into the 5' end of the HIV-1 Gag coding sequence [1]. Interactions of the Gag polyprotein with stem-loops 2, 3, and 4 ensure efficient encapsidation of the gRNA [1]. Nonetheless, a fragment that included the ψ region did not confer packaging of a heterologous RNA [2]. This is in contrast to the murine leukemia virus (MLV) gRNA which contains a defined *cis *element of ~175 bp in its 5' UTR capable of packaging heterologous RNAs into MLV derived viral particles [3,4]. These data indicate that the HIV-1 packaging system is more complex than that of MLV, comprising multiple *cis *elements, some of which are outside of the canonical packaging signal. A number of loss-of-function studies demonstrated that additional *cis *elements throughout the 5' UTR (R, U5, and PBS) also impact encapsidation of gRNA [5-8].

HIV-1 *cis *and *trans *components influence gRNA encapsidation through RNA-RNA and RNA-protein interactions occurring within the nucleus and cytoplasm, prior to localization of Gag-RNA complexes to sites of particle assembly at the plasma membrane. Cytoplasmic interactions, such as RNA-RNA dimerization [9] and Gag-RNA interactions [10], are critical steps in the encapsidation mechanism, but are downstream steps to the export of RNA from the nucleus to the cytoplasm. The HIV-1 Rev protein mediates nuclear export of unspliced full-length, as well as partially spliced, viral RNAs by bridging an interaction between the Rev response element (RRE) in the gRNA and the host cell CRM1 nuclear export pathway [11]. The Rev/RRE system is highly conserved among lentiviruses; a mechanism not shared by simple retroviruses such as MLV, which accomplish RNA nuclear export independent of viral encoded proteins [12,13]. Accumulating evidence indicates that the Rev/RRE system also contributes to translation and RNA encapsidation events that occur in the cytoplasm, after nuclear export [14]. HIV-1 Rev was shown to enhance translation 1-3 orders of magnitude, concurrent with nominal changes in cytoplasmic RNA levels [15]. In the context of HIV-1 virus, and viral vectors, the Rev/RRE was also identified to influence RNA encapsidation into viral particles [16-19]. The HIV-1 Rev protein is not known to be a constituent of the viral particle, thus, conceivably, Rev may influence gRNA encapsidation at a step prior to, or coincident with the Gag-RNA interaction, and consequently incorporation of the gRNA into a viral particle. As alluded to above, disparate studies indicate that efficient and specific RNA encapsidation into HIV-1 viral particles rely upon multiple components comprised of both *cis *elements in the RNA (R, U5, PBS, ψ, and RRE) and viral encoded *trans *factors (Gag protein and Rev). However, it is not understood if these components can function independently of each other, or if the RNA encapsidation mechanism is a single pathway relying upon the concerted effects of the various components.

We reasoned that the HIV-1 Rev/RRE system and *cis *elements in the 5' UTR may function within the same pathway, thus the combined effects may be necessary for efficient and specific encapsidation of RNA into HIV-1 viral particles. Mutational analysis, in the innate viral context, has commonly been used to address the contributions of the abovementioned *cis *elements and *trans *factors to encapsidation [1]; however, difficulty in parsing pleiotropic functions may confound effects attributed to encapsidation. A system that relies upon gain-of-function may be suitable for discerning the impact of multiple viral components on encapsidation. Here, the Rev/RRE system and 5' UTR *cis *elements were systematically reconstructed in the middle of a simple retroviral vector RNA (derived from MLV) to investigate the collective, and independent, impact on the gain of encapsidation function into HIV-1 derived viral particles. We show that i) the Rev/RRE system can augment encapsidation of MLV vector RNA independent of the canonical HIV-1 packaging signal; ii) the Rev/RRE system is required for *cis *elements from the 5' UTR to mediate efficient and specific encapsidation into HIV-1 viral particles; iii) a functional packaging system is composed of multiple components (including 5' UTR *cis *elements, nucleocapsid, and Rev/RRE system); iv) the Rev/RRE system and 5' UTR *cis *elements synergize to increase vector titers that rival those of HIV-1 derived vectors; and v) HIV-1 delivered heterologous RNAs render episomes (predominantly 1-LTR circles) in transduced cells that may prove beneficial as non-integrating vectors in gene therapy protocols.

## Results

### HIV-1 Rev augments encapsidation of a heterologous RNA independent of the canonical HIV-1 packaging signal

**Figure 1:**
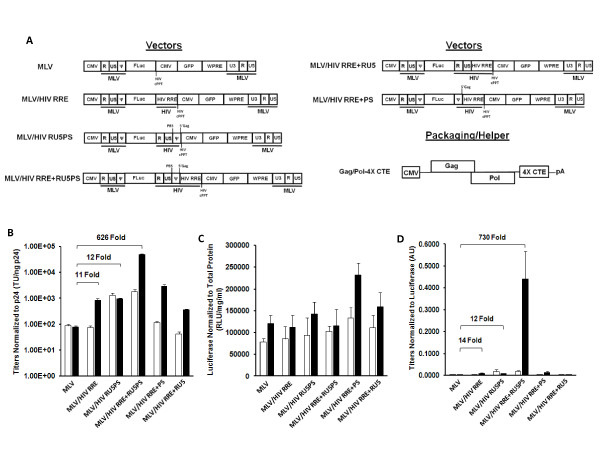
**HIV-1 Rev/RRE system and *cis *elements in the 5'UTR augment vector titers. A**. Full-length MLV/HIV chimeric vector RNAs are expressed from a CMV (cytomegalovirus) promoter in transfected 293T cells. MLV and HIV *cis *elements can be distinguished by black underscore. Chimeric vector names are represented as MLV/HIV followed by corresponding HIV *cis *elements incorporated: RRE (Rev Response Element), R (repeat), U5 (unique region 5), PS (packaging signal comprised of ψ [canonical packaging signal and into 5' Gag region]), cPPT (central polypurine tract), PBS (primer binding site). Also incorporated are the WPRE (woodchuck hepatitis virus post-transcriptional regulatory element), FLuc (firefly luciferase gene), and GFP (green fluorescent protein gene). HIV-1 Gag-Pol 4X CTE helper construct was used to express structural and enzymatic proteins to generate viral particles independent of HIV-1 Rev protein. **B**. Vector titers normalized to p24 are shown in the absence (white bars) and presence (black bars) of Rev. The influence of adding HIV-1 *cis *elements to the MLV vector is indicated by fold increases in the presence of Rev relative to the standard MLV vector. Fold increases for MLV/HIV RRE + PS (38 fold) and MLV/HIV RRE + RU5 (5 fold) are not indicated on the graph. **C**. Luciferase levels normalized to total protein are shown for each vector. **D**. Titers expressed as a ratio to luciferase are shown as arbitrary units (AU). Fold increases for MLV/HIV RRE + PS (22 fold) and MLV/HIV RRE + RU5 (4 fold) are not indicated on the graph. Error for all bar graphs is expressed as ±S.D. All experiments were performed in triplicate.

**Figure 2:**
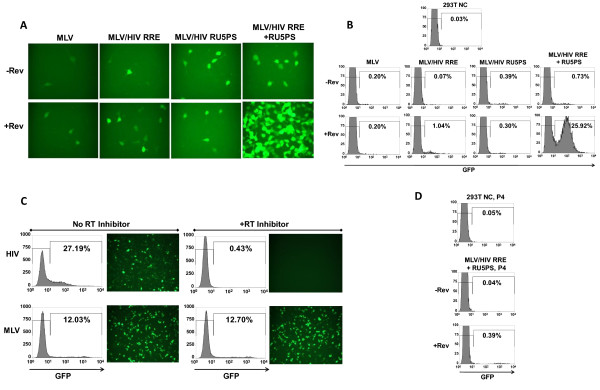
**Transduction of 293T cells with chimeric MLV/HIV vectors packaged into HIV-1 viral particles**. **A and B**. 293T cells were transduced with equivalent amounts of p24 capsid protein (50 ng), as determined for each of the indicated chimeric vectors. The influence of the HIV-1 Rev/RRE system, and 5' UTR *cis *elements, on transduction was assessed by fluorescence microscopy **(A) **and FACscan analysis **(B) **at 7 days post-transduction. **C**. 293T cells were transduced in the absence (No RT Inhibitor), or presence (+RT Inhibitor), of the HIV-1 specific non-nucleoside reverse transcriptase inhibitor, etravirine (100 nM). Transduced cells were assessed by fluorescence microscopy and FACscan analysis. **D**. The capacity of the MLV/HIV RRE + RU5PS vector to be stably maintained after 4 cell passages was examined by FACscan analysis. The percent GFP positive cells are indicated for each FACscan and 293T negative control (NC) cells are shown.

The Rev/RRE system was recently demonstrated to enhance encapsidation of a HIV-1 vector RNA into HIV-1 viral particles [17]. In this previous work, however, the Rev/RRE system was examined in the context of HIV-1 vectors that contain *cis *elements from the 5' UTR including R, U5, PBS, and ψ regions that may also have contributed to the enhanced encapsidation. Additionally, the combination of the Rev/RRE system and *cis *elements from the 5' UTR may have pleiotropic effects which impact other stages of the viral life cycle (i.e. reverse transcription [20]). To isolate encapsidation effects directly attributable to the Rev/RRE system we assembled a heterologous RNA system derived from murine leukemia virus (MLV) vector RNA (Figure 1A). Nuclear export of MLV vector RNA is autonomous [12,13], a feature exploited to elucidate the effects of the Rev/RRE on HIV-1 processes (i.e. encapsidation). Heterologous vector RNAs were packaged into viral particles generated from a helper system (Gag/Pol-4X CTE) that does not rely upon the Rev/RRE system for nuclear export of RNA encoding the structural and enzymatic proteins (Figure 1A). By circumventing dependence upon Rev for nuclear export of *gag/pol *RNA, and vector RNA, we could directly analyze the effect of Rev on encapsidation. The MLV/HIV RRE chimeric vector was constructed to express enhanced green fluorescent protein (eGFP) from an internal CMV promoter to provide an indirect indication of the RNA incorporated into viral particles (Figure 1A). Vector titers were determined by scoring for GFP expression following transduction of 293T cells, and normalized to amounts of p24 capsid protein (Figure 1B). Incorporation of only the HIV-1 RRE into the heterologous MLV vector (MLV/HIV RRE vector) enhanced titers 11-fold in the presence of Rev. This is the first report that the Rev/RRE system can influence packaging of a foreign RNA into HIV-1 viral particles. These results indicate that the HIV-1 Rev/RRE system can impact titers of vectors devoid of HIV-1 *cis *elements known to affect RNA packaging. Furthermore, the Rev/RRE system conferred corresponding increases in transduction of 293T cells exposed to equivalent amounts of p24 capsid protein (Figure 2A and 2B), demonstrating that the Rev mediated increase in titer cannot be attributed to Rev effects on HIV-1 particle production. Since it is well established that the Rev protein enhances nuclear export of RNAs containing a RRE, observed increases in titers may be a consequence of augmented nuclear export. This possibility was investigated by measuring the cytoplasmic vector levels in the producer cells.

The vectors were configured to indirectly assess cytoplasmic levels of vector length RNAs during production by situating the firefly luciferase gene such that it was included in the full-length vector RNA, but not in RNAs expressed from the internal promoter (Figure 1A). Luciferase expression in the 293T producer cells is indirect evidence for cytoplasmic RNA, and may be subject to translational influences. Luciferase levels were marginally affected by Rev, indicating that Rev did not influence nuclear export of the MLV/HIV RRE vector (Figure 1C). Examining the titer/luciferase ratio (Figure 1D) revealed that the effects of the Rev/RRE system primarily alter vector titers (14 fold) with minimal cytoplasmic changes. Reasoning that the Rev/RRE system may mediate packaging of vector RNA into HIV-1 viral particles, we were encouraged to further explore the mechanism mediating RNA encapsidation.

**Figure 3:**
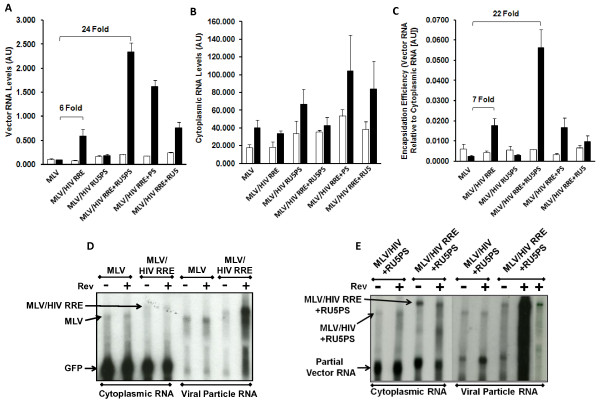
**HIV-1 Rev/RRE and *cis *elements in the 5'UTR cooperatively enhance RNA encapsidation into HIV-1 viral particles. A**. Vector RNA was measured by qRT-PCR and expressed in arbitrary units (AU). RNA levels for all graphs are shown in the absence (white bars) and presence (black bars) of Rev. The influence of adding HIV-1 *cis *elements to the MLV vector is indicated by fold increases in the presence of Rev relative to the standard MLV vector. Fold increases in vector RNA for MLV/HIV RU5PS (1.9 fold), MLV/HIV RRE + PS (17 fold) and MLV/HIV RRE + RU5 (7.7 fold) are not indicated on the graph. **B**. Cytoplasmic RNA was isolated from vector producer 293T cells at the time of vector harvesting. Relative RNA levels were obtained and recorded as done for vector RNA in part A. **C**. Efficiency of encapsidating RNA into HIV-1 viral particles is expressed as a ratio of vector RNA in viral particles to cytoplasmic RNA available for encapsidation. Relative levels are expressed like vector RNA in part A. Fold increases for MLV/HIV RU5PS (1.2 fold), MLV/HIV RRE + PS (6.7 fold) and MLV/HIV RRE + RU5 (3.9 fold) are not indicated on the graph. **D**. Northern blot analysis of cytoplasmic and vector RNA isolated from MLV and MLV/HIV RRE in the absence (-) and presence (+) of Rev. Vector length RNA species were detected with a GFP labeled probe, as well as an additional RNA species (labeled GFP) generated from the internal CMV promoter. **E**. Northern blot analysis of cytoplasmic and vector RNA isolated from MLV/HIV RU5PS and MLV/HIV RRE + RU5PS in the absence (-) and presence (+) of Rev. Vector length RNA species were detected with a probe to a region in the 5' end of the vector, as well as an additional RNA species (labeled 'partial vector RNA'). Cytoplasmic and vector RNAs are shown at different exposures of the same blot. Last lane (far right) is a shorter exposure of adjacent left lane. Error for all bar graphs is expressed as ±S.D. All experiments were performed in triplicate.

Encapsidation efficiency is a measure of RNA packaged into viral particles relative to the RNA available for packaging, in the cytoplasm. Vector RNA was isolated from viral particles in the media of 293T producer cells and vector producing cells were fractionated to obtain cytoplasmic RNA. Cytoplasmic separation was routinely monitored using western blot analysis for the absence of the nuclear specific protein, nucleolin, from the cytoplasm (Additional file 1, Figure S1A). Relative RNA levels in viral particles and cytoplasm of producer cells were examined by qRT-PCR and northern blot analysis (Figure 3). In the absence of HIV-1 5' UTR *cis *elements Rev enhanced the levels of MLV/HIV RRE vector RNA in viral particles 6 fold compared to the MLV vector (Figure 3A), which is in line with what was observed for the titers (Figure 1B). Compared to the basic MLV vector the Rev/RRE system did not impact cytoplasmic vector RNA levels (Figure 3B), thus the increase in RNA encapsidation is similar to that observed in viral particles, 7-fold (Figure 3C). Northern blot analysis supported the observation that the Rev/RRE system could augment encapsidation independent of additional HIV-1 5' UTR *cis *elements (Figure 3D). Specificity is also apparent from northern blot analysis, demonstrating that vector length RNA species is more efficiently packaged in the presence of Rev than a smaller RNA species (labeled as GFP generated from the internal promoter) lacking the RRE *cis *element. Overall, our data demonstrate that the Rev/RRE system can enhance encapsidation of a heterologous RNA into HIV-1 viral particles independent of the canonical HIV-1 packaging signal, as well as other 5' UTR *cis *elements. These data indicate that the Rev/RRE system may be a central component of a RNA encapsidation mechanism that is traditionally associated with *cis *elements in the 5' UTR (R, U5, PBS, and ψ). We examined the proposition that an encapsidation mechanism is the concerted effect of all these components.

### HIV-1 Rev/RRE system is required for the 5' UTR *cis *elements to mediate efficient RNA encapsidation

We have demonstrated that the Rev/RRE system is a part of the encapsidation mechanism. However, it is well established that encapsidation is primarily mediated through *cis *elements in the 5' UTR, predominantly protein-RNA interactions of the nucleocapsid with ψ [10], and RNA-RNA dimerization via stem-loop 1 of ψ [9]. The HIV-1 Rev/RRE, as well as the aforementioned protein-RNA and RNA-RNA interactions may comprise a *bona fide *packaging system that may be defined by the capacity to support efficient and specific encapsidation of a heterologous RNA into HIV-1 derived viral particles. To characterize the role of the Rev/RRE in the context of a packaging system that comprises the 5' UTR *cis *elements we generated a series of heterologous MLV/HIV vectors (Figure 1A). The entire 5' UTR was either independently incorporated into the heterologous MLV RNA (MLV/HIV RU5PS), or inserted in the context of the RRE (MLV/HIV RRE + RU5PS). Since *cis *elements in the R and U5 regions have also been shown to impact encapsidation [5-8], the 5' UTR was also further separated into the R/U5 (MLV/HIV RRE + RU5) and ψ (MLV/HIV RRE + PS) in the context of the RRE (Figure 1A). Each of the vectors was titered on 293T cells by scoring for GFP positive cells, and normalizing to levels of p24 capsid protein.

The complete contigent of *cis *elements from the 5' UTR (MLV/HIV RU5PS) moderately enhanced titers (12 fold) independent of Rev, whereas in the context of the RRE (MLV/HIV RRE + RU5PS) Rev dramatically augmented titers (626 fold) compared to the basic MLV vector (Figure 1B); an effect that was visually, and quantitatively, outstanding upon transduction of 293T cells with equivalent amounts of p24 capsid protein (Figure 2A and 2B). Notably, titers of the MLV/HIV RRE + RU5PS vector were 1.07 × 10^7 ^TU/ml, which is in the 10^7^-10^8 ^TU/ml range of those obtained with standard HIV-1 vectors prior to concentration; a comparison that was also observed after normalization to p24. These data indicate that the Rev/RRE system and 5' UTR *cis *elements synergize to achieve the increase in vector titer. Further separation of the 5' UTR *cis *elements into the canonical packaging signal (MLV/HIV RRE + PS) or R/U5 (MLV/HIV RRE + RU5) did not achieve levels of titer similar to the vector containing the entire 5' UTR and RRE (Figure 1B), demonstrating the significance of retaining a fully intact 5' UTR. Furthermore, to confirm that GFP titers are a result of reverse transcription of heterologous MLV vector RNA by the HIV-1 reverse transcriptase, transduction of 293T cells was assessed by FACs analysis and fluorescence microscopy following treatment with the HIV-1 specific non-nucleoside RT inhibitor, etravirine (Figure 2C). Etravirine specifically inhibited transduction of the MLV/HIV RRE + RU5PS vector packaged into HIV-1 viral particles, but did not inhibit transduction of the same vector packaged into MLV viral particles (Figure 2C). These data also indicate that GFP expression is not a consequence of pseudotransduction. Although Rev enhanced titers of all the above mentioned vectors containing the RRE, cytoplasmic luciferase levels remained relatively similar, indirectly indicating that increased titers were probably not a consequence of increased nuclear export of vector RNA (Figure 1C and 1D). The titers were a clear indication that a comprehensive HIV-1 packaging system may comprise the synergistic influences of the Rev/RRE system and *cis *elements from the 5' UTR, as well as nucleocapsid protein. Considering this possibility, we sought to directly characterize the Rev impact on the encapsidation efficiency of heterologous RNAs, containing *cis *elements from the 5' UTR, into HIV-1 viral particles. Quantitative RT-PCR was employed to quantify vector RNA in viral particles, and in producer cells.

Surprisingly, as shown in Figure 3 A and 3C, in contrast to the increase in vector titers (Figure 1B), incorporation of the 5' UTR *cis *elements into the heterologous MLV vector (MLV/HIV RU5PS) did not enhance RNA encapsidation in either the absence or presence of Rev. Most importantly, in the context of the RRE (MLV/HIV RRE + RU5PS), however, the 5' UTR *cis *elements exhibited a 22 fold increase in heterologous vector RNA encapsidation into HIV-1 viral particles in the presence of Rev (Figure 3C). Rev-dependent encapsidation was clearly a consequence of enhanced RNA packaged into viral particles (Figure 3A), not increases in cytoplasmic RNA (Figure 3B). Notably, however, the cytoplasmic levels of RNA may vary between experiments, which may be partially due to transfection variation (Additional file 1, Figure S1 B-D). Nonetheless, transfection would not impact encapsidation measurements which are derived from the ratio of vector RNA in the viral particles relative to vector RNA in the cytoplasm. These data demonstrate that the Rev-RRE interaction may initially be required to render the RNA amenable for subsequent steps in the encapsidation mechanism that conventionally involve *cis *elements from the 5' UTR, such as interaction between nucleocapsid and the canonical packaging signal. Moreover, the enhanced encapsidation effect of the MLV/HIV RRE + RU5PS chimeric vector is dependent upon the complete contingent of 5' UTR *cis *elements, since dissection of the 5' UTR *cis *elements into the canonical packaging signal (MLV/HIV RRE + PS) or RU5 (MLV/HIV RRE + RU5) did not result in corresponding increases in encapsidation efficiency, in the presence of Rev, which was comparable to the MLV/HIV RRE vector (Figure 3C). The Rev-dependent enhancement of MLV/HIV RRE + RU5PS vector RNA encapsidation obtained by qRT-PCR was bolstered by northern blot analysis showing strong Rev-dependent increase in levels of RNA encapsidated into HIV-1 viral particles, despite nominal changes in cytoplasmic vector RNA levels (Figure 3E). Notably, within the cytoplasmic RNAs our probe detects a dominant smaller species of vector RNA (termed 'partial vector RNA'). Although the full-length vector RNA is present at lower levels in the cytoplasm, the Rev/RRE system and 5' UTR *cis *elements impart the ability of the full-length vector RNA to out compete the more abundant 'partial vector RNA' species for packaging into viral particles (Figure 3E); demonstrating the specificity that these components confer upon a RNA for encapsidation.

Overall, our data demonstrate that: i) Rev is required for efficient encapsidation of a heterologous RNA that is subsequently mediated by RNA-RNA and RNA-protein interactions through *cis *elements in the 5' UTR; ii) *cis *elements from the 5' UTR exhibit effects that can enhance heterologous vector titer without increasing RNA encapsidation; and iii) a packaging system competent for heterologous RNA encapsidation should minimally include the Rev/RRE system and all *cis *elements of the 5' UTR.

### The HIV-1 Rev/RRE and 5' UTR *cis *elements do not augment RNA encapsidation into MLV derived viral particles

**Figure 4:**
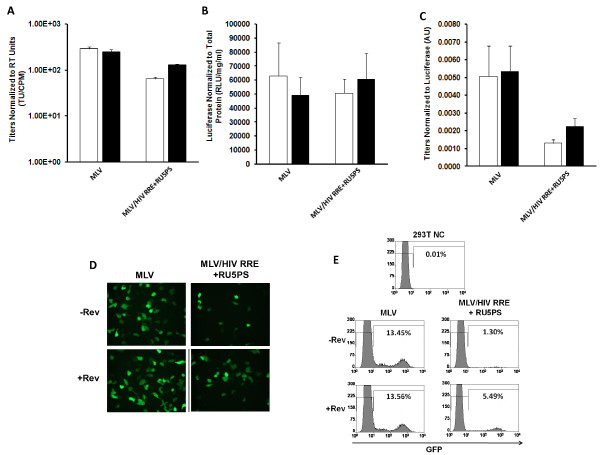
**HIV-1 Rev/RRE and *cis *elements in the 5'UTR do not influence vector titers after packaging into MLV viral particles. A**. Titers of MLV/HIV chimeric vectors were obtained by scoring for GFP positive cells following transduction of 293T cells. Titers are expressed as transducing units (TU) normalized to the amount of RT units (counts per minute [CPM]). **B**. Normalized luciferase levels were determined in transfected 293T producer cells. Luciferase levels were normalized to total cell protein. **C**. Titers (part A) expressed as a ratio to levels of luciferase (part B) shown in arbitrary units (AU). All experiments were executed in the absence (white bars) and presence (black bars) of Rev. Error for all bar graphs is expressed as ±S.D. All experiments were performed in triplicate. **D **and **E**. 293T cells were transduced with equivalent amounts of RT units (6 × 10^5 ^CPM), as determined for each of the indicated chimeric vectors. The influence of the HIV-1 Rev/RRE system, and 5' UTR *cis *elements, on transduction was assessed by fluorescence microscopy **D **and FACscan analysis **E **at 7 days post-transduction. The percent GFP positive cells are indicated for each FACscan.

Manipulating the HIV-1 packaging system, described above, to efficiently encapsidate a foreign RNA into HIV-1 viral particles may be a general biological phenomenon that can be exploited for RNA encapsidation into non-HIV-1 viral particles. The heterologous MLV RNA vector system was used to examine if the HIV-1 Rev/RRE and 5' UTR *cis *elements can mediate packaging of RNA into MLV viral particles, thereby resulting in a loss of specificity for HIV-1 viral particles. Retention of the MLV packaging signal bestows dual functionality onto the MLV/HIV RRE + RU5PS chimeric vector RNA to also allow for packaging into MLV derived viral particles. Accordingly, this vector was used to examine if the HIV-1 Rev/RRE and 5' UTR *cis *elements alter the encapsidation efficiency into MLV viral particles. Direct comparison of the chimeric vector to the basic MLV vector revealed that there was little impact of the Rev/RRE system on normalized titers or luciferase levels (Figure 4A and 4B). In fact, transduction with equivalent units of MLV reverse transcriptase resulted in a reduced number of transduced cells, compared to the basic MLV vector (Figure 4D and 4E). Moreover, the titers were slightly lower than those of the basic MLV vector, resulting in decreased levels after normalization to luciferase (Figure 4C). In contrast to HIV-1 viral particles, these data indicated that the HIV-1 Rev/RRE and 5' UTR *cis *elements do not influence the packaging of RNA into MLV viral particles.

**Figure 5:**
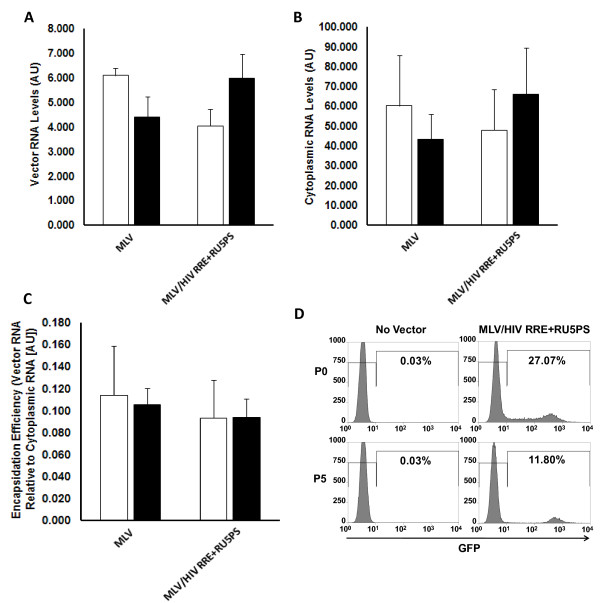
**HIV-1 Rev/RRE and *cis *elements in the 5' UTR do not augment RNA encapsidation into MLV viral particles**. **A**. Vector RNA packaged into MLV derived viral particles was isolated from equivalent amounts of RT units in the media of 293T producer cells. RNA levels were measured by qRT-PCR and are expressed as arbitrary units (AU). RNA levels are shown in the absence (white bars) and presence (black bars) of Rev. **B**. Cytoplasmic RNA was isolated from vector producer cells coincident with harvesting vector particles. Relative levels are expressed similar to vector RNA in part A. **C**. Efficiency of encapsidating RNA into MLV viral particles is expressed as a ratio of vector RNA in viral particles to cytoplasmic RNA available for encapsidation. **D**. Transduction of 293T cells with MLV/HIV RRE + RU5PS at 5 days post-transduction (no passaging of cells, P0), and after 5 passages of cells (P5). Percent GFP positive cells were assessed by FACscan analysis and compared to non-transduced (No Vector) 293T cells. Error for all bar graphs is expressed as ±S.D. All experiments were performed in triplicate.

Investigation of the encapsidation efficiency into MLV viral particles exposed a picture similar to that obtained with the titer/luciferase assays. There was no effect of the Rev/RRE and 5' UTR *cis *elements on levels of vector RNA in MLV viral particles, or in the producer cell cytoplasm (Figure 5A and 5B). Consequently, these HIV-1 components also had no effect on RNA encapsidation into MLV viral particles (Figure 5C). These results imply that the Rev/RRE system and 5' UTR *cis *elements confer specificity onto the heterologous MLV vector RNA for encapsidation into HIV-1 viral particles, but provide no advantage for encapsidation into MLV viral particles. Using a single RNA system with different packaging specificities, we were able to demonstrate that HIV-1 and MLV commandeer distinct mechanisms to select vector RNAs from the milieu of host cell RNAs and promote RNA encapsidation into nascent viral particles.

### Packaging chimeric MLV/HIV vector RNAs into HIV-1 viral particles reveals unique transduction properties

**Figure 6:**
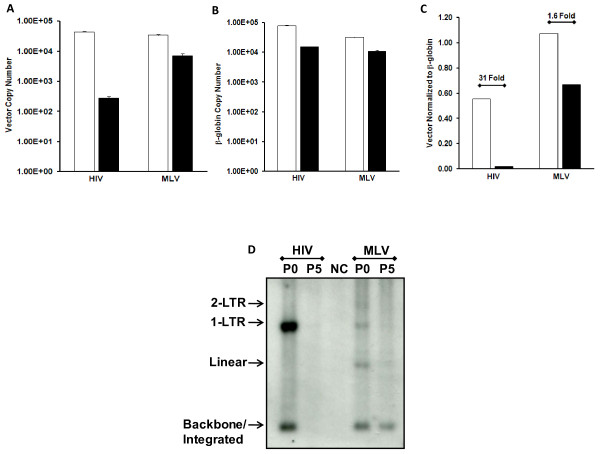
**Heterologous MLV RNAs form, predominantly, 1-LTR episomal cDNAs following delivery with HIV-1 viral particles**. MLV/HIV RRE + RU5PS chimeric vector was packaged into HIV-1 and MLV viral particles in the presence of Rev. 293T cells were transduced with equivalent transducing units for HIV-1 and MLV packaged vectors. Total cellular DNA was harvested at 5 days post-transduction (episomal and integrated vector DNA, P0), and after five cell passages (integrated vector DNA, P5). Vector DNA copy number as measured by qPCR to the WPRE **A**, and β-globin DNA copy number **B**, were determined by qPCR. Data are shown without cell passages (P0; white bars) and after 5 cell passages (P5; black bars). Vector DNA copy number was normalized to β-globin copy number **C**, and fold decreases in vector DNA levels, after passaging cells, are shown. Error for all bar graphs is expressed as ±S.D. **D**. Southern blot analysis of total DNA isolated from 293T cells transduced with MLV/HIV RRE + RU5PS vector packaged into either HIV, or MLV, viral particles (as indicated above lanes). Total DNA was isolated at 5 days posttransduction (P0) and after 5 passages of cells (P5). DNA was digested to distinguish between 2-LTR, 1-LTR, and linear episomal forms, as well as the vector backbone which is indicative of integrated vector DNA after passaging cells.

Depending on the type of viral particle, MLV or HIV-1, carrying the vector RNA, a different transduction profile may be anticipated. In the context of MLV viral particles the chimeric vector was retained after multiple cell passages to eliminate episomal vector DNA species, as indicated by FACs analysis of GFP positive cells (Figure 5D). In contrast, GFP positive cells from transduction with HIV-1 delivered chimeric vector were almost completely eliminated (Figure 2B and 2D), indicating that GFP may be expressed from episomal DNA vector forms not competent for integration. The presence of total episomal DNA forms was assessed by qPCR for vector copy number (Figure 6A) relative to copy number of β-globin (Figure 6B). In line with GFP results, qPCR quantitation of total vector DNA before and after passaging cells yielded a >30 fold decrease in vector DNA after passaging 293T cells transduced with HIV-1 particles, compared to <2 fold decrease from MLV viral particles (Figure 6 C). MLV/HIV chimeric vector RNA delivered by HIV-1 viral particles primarily cedes proviral episomal DNA forms that can be composed of 1-LTR, 2-LTR, linear, or mutant forms.

Southern blot analysis revealed that the HIV-1 packaged MLV/HIV RRE + RU5PS vector exists predominantly as a 1-LTR episome that can be diluted following multiple cell passages (Figure 6D). In contrast, the same vector delivered with MLV viral particles exhibited a very different profile, primarily as integrated DNA accompanied by minimally detectable levels of linear, 1-LTR, and 2-LTR episomes (Figure 6D). Reverse transcription of the chimeric vector RNA by HIV-1 RT clearly leads to dominant 1-LTR episomal species that are apparently responsible for the observed GFP expression, albeit expression is extremely low. The chimeric vector packaged into HIV-1 viral particles curtails the presence of linear episomal forms that may be substrates for illegitimate integration as described earlier by Kantor *et al*. [21], as well as nonhomologous integration at sites of strand breakage in the host cell genome. A non-integrating vector that minimizes perturbations of the host cell genome would be most desirable for gene therapy protocols.

**Figure 7:**
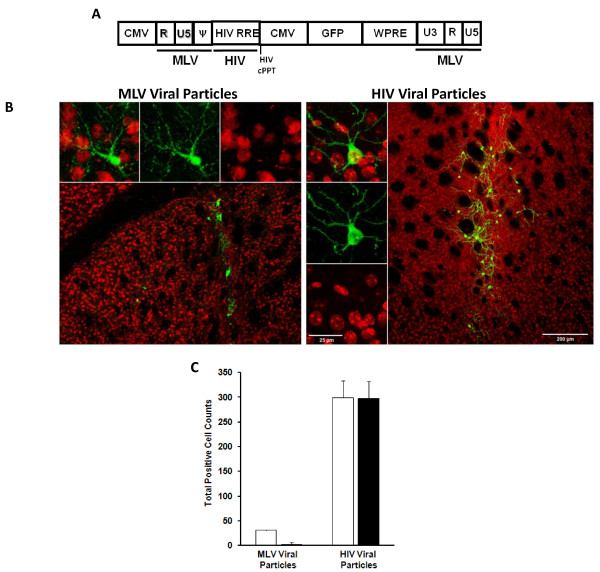
**HIV-1 structural and enzymatic proteins can deliver a heterologously packaged RNA to non-dividing cells *in vivo***. **A**. MLV/HIV RRE chimeric vector used to investigate transduction of mouse neurons *in vivo *following packaging into MLV or HIV-1 viral particles. **B**. Mouse brains were injected into the striatum with equivalent transducing units of MLV/HIV RRE vector packaged into either MLV (left panels), or HIV-1 (right panels), derived viral particles. Brain sections were imaged by confocal microscopy following co-staining for neurons (NeuN, red) and vector particles (GFP, green). Images depicting both vector and neurons can be seen for MLV (top left and low magnification) and HIV (left top and low magnification) viral particles. Independent images of vector and neurons are shown for MLV (top middle and top right, respectively) and HIV (left middle and left bottom, respectively) viral particles. **C**. The graph represents the total number of cells scored for GFP only (white bars), and colocalized GFP + NeuN (black bars) from 5 mice in each group. Error for bar graph is expressed as ±S.D.

Unique to lentiviral vectors is the ability to establish stable transgene expression in non-dividing cells. HIV-1 mediated delivery of a MLV/HIV RRE chimeric vector that expresses GFP (Figure 7A) demonstrated efficient transduction of mouse brain neurons in the striatum (Figure 7B and 7C). HIV-1 delivered vector (GFP marker) colocalized in neurons stained for the neuronal nucleus (NeuN) marker, whereas the same vector delivered with MLV viral particles did not exhibit neuronal transduction (Figure 7B); an observation that was consistent over multiple brain sections (Figure 7C). Nonetheless, MLV delivered vector did yield a moderate number of GFP positive cells that were not NeuN positive and appeared to be morphologically distinct. These data are a clear indication that heterologous MLV vector RNAs, packaged into HIV-1 viral particles, can assume transduction properties dictated by HIV-1 structural and enzymatic proteins, with the exception that reverse transcribed cDNAs are not competent for HIV-1 mediated stable integration.

## Discussion

The objective of this study was to understand the role of the HIV-1 Rev/RRE system in the sophisticated mechanism of selecting a RNA species from the milieu of host cell RNAs for efficient and specific encapsidation into HIV-1 viral particles. Rev/RRE-dependent encapsidation was specifically assessed through reconstruction of the HIV-1 packaging system in the context of a heterologous MLV vector RNA, thereby averting the RNA nuclear export effects of the Rev/RRE system. We have shown for the first time that the Rev/RRE system can enhance RNA encapsidation of a heterologous RNA into HIV-1 viral particles in the absence of more conventional *cis *packaging elements localized in the 5' UTR of HIV-1 RNA (Figure 3). Most importantly, the HIV-1 Rev/RRE system was required for *cis *elements in the 5' UTR to mediate efficient encapsidation into HIV-1 viral particles. Furthermore, prior mutagenesis studies have implicated 5' UTR *cis *determinants adjacent to the canonical packaging signal as important for efficient RNA encapsidation through loss of function analysis [5-8]. Our data expand this view by demonstrating that the 5' UTR *cis *elements are not separable to achieve efficient encapsidation of heterologous RNAs (Figure 3).

Our results build upon previous work demonstrating a role for Rev in RNA encapsidation [17,18,22-24]. Conceivably, the Rev/RRE may impact the cytoplasmic distribution of RNA without direct involvement in packaging RNA into viral particles at the plasma membrane, a view consistent with the inability to detect HIV-1 Rev in viral particles [14]. Additionally, the Rev/RRE system may alter the conformation of the 5' UTR creating a context that is more receptive to an interaction with HIV-1 Gag, and subsequent packaging. Recent *in vitro *evidence indicates that HIV-1 Rev can influence translation in a concentration dependent manner that does not rely upon the RRE, but rather an interaction between Rev and a *cis *determinant in the 5' UTR [25]. At moderate concentrations Rev enhanced translation, but at high concentrations translation was inhibited [25]. The same interaction was also implicated in RNA encapsidation [18], therefore it is plausible that Rev may promote RNA packaging at high concentrations, possibly acting as a "switch" between translation and encapsidation. Nonetheless, experiments supporting such a mechanism are still required.

The requirement for the HIV-1 Rev/RRE system in the encapsidation mechanism implies that the Rev/RRE may confer specificity onto HIV-1 RNA during the initial steps of the mechanism when Rev interacts with RNA in the nucleus. Such a mechanism might ensure early selection of viral RNA from the milieu of host cell RNAs, concomitant with transcription in the nucleus. Our data support a role for the Rev/RRE system in conferring specificity of RNA packaged into HIV-1 viral particles (Figure 3 and 5). The HIV-1 Rev/RRE did not confer an encapsidation advantage of the heterologous RNA into MLV viral particles; consistent with the notion that HIV-1 and MLV utilize distinct mechanisms to encapsidate viral RNA [26,27]. In the absence of the HIV-1 Rev/RRE, MLV vector RNA exhibited no added specificity upon packaging into HIV-1 viral particles, in agreement with a study showing that a MLV vector RNA was not enriched in HIV-1 viral particles relative to cellular mRNAs [28]. Nonspecific (yet measurable) packaging of MLV RNA raise the concern that stable HIV-1 packaging cell lines, generated by introducing HIV-1 *gag *and *pol *genes with MLV vectors [29,30], have the potential to package, transfer, reverse transcribe, and recombine HIV-1 *gag *and *pol *genes in recipient cells.

Although our system revealed an essential role for the Rev/RRE system in efficient and specific RNA encapsidation into HIV-1 viral particles, we observed a discrepancy between the efficiency of RNA encapsidation (Figure 3) and p24 normalized titers (Figure 1) for heterologous vector RNAs harboring the entire 5' UTR. Inclusion of the HIV-1 primer binding site (PBS) and flanking 5' UTR sequences might accommodate more efficient reverse transcription and transduction of the heterologous RNA, yielding higher titer in the form of GFP positive cells. Efficient reverse transcription from the HIV-1 PBS is accomplished by the specific packaging of tRNA^Lys ^primers into HIV-1 viral particles [20]. Furthermore, the tRNA^Lys ^primers may also promote reverse transcription at a low efficiency from the MLV PBS (normally primed by tRNA^Pro^) in the heterologous MLV vectors that lack a HIV-1 PBS; a rationalization consistent with a study showing that, although impaired, HIV-1 replication was retained if the HIV-1 PBS was altered to that of MLV [31].

The heterologous RNA packaging system yields transduction efficiencies comparable to those of standard HIV-1 vectors. The unique capacity of the heterologous vector to remain episomal exposes it to manipulation for therapeutic gene delivery purposes. Episomal vectors have recently been sought as safer alternatives to integrating lentiviral vectors for gene therapy protocols requiring transient gene expression in dividing cells, or long-term expression in non-dividing cells [32,33]. HIV-1 delivery of the chimeric heterologous vector resulted in the dominant formation of 1-LTR episomal forms (Figure 6), which was indicative of alterations in reverse transcription. Absence of a HIV-1 3'PPT, 5'PBS, and *att *sites may obviate the generation of linear cDNA episomes during reverse transcription, which are precursors to 2-LTR circles and integration competent linear cDNA; consistent with a recent report showing that deletion of the HIV-1 3' PPT encourages 1-LTR circle formation at the expense of linear forms [21]. Gene expression from the chimeric MLV/HIV system remains a challenge, yet, like non-integrating HIV-1 vectors [21,34], expression is detectable following *in vivo *transduction of neurons in the mouse brain striatum (Figure 7). Restricting the synthesis of linear episomes through the use of the chimeric MLV/HIV vector system may impart improved safety benefits, over conventional non-integrating HIV-1 vectors, by reducing illegitimate integration.

## Conclusions

Using a heterologous RNA system, we were able to isolate the encapsidation and transduction effects of different HIV-1 *cis *and *trans *components. Most importantly, however, these studies revealed the concerted effects of multiple HIV-1 components through gain-of-function studies. Conventional loss-of-function studies have implicated several of the aforementioned HIV-1 *cis *and *trans *components in the encapsidation mechanism, but do not reveal the interdependence of these components. Our data demonstrate that the HIV-1 Rev/RRE system is essential for *cis *elements in the 5' UTR (including the canonical packaging signal) to mediate efficient and specific encapsidation of a heterologous RNA into HIV-1 viral particles. Moreover, the Rev/RRE system could augment RNA encapsidation independent of all *cis *elements from the 5' UTR. Therefore, we believe that, in addition to its traditional role in nuclear export, the Rev/RRE system may have a critical role in making the HIV-1 RNA more amenable to RNA-RNA and RNA-protein interactions in the cytoplasm, which support subsequent RNA encapsidation. Nonetheless, the combined effects of the Rev/RRE system and 5' UTR *cis *elements are not limited to encapsidation since these components can synergize to yield transduction efficiencies that approach those of standard HIV-1 vectors. The unique transduction properties (1-LTR episomes) associated with heterologous RNA, delivered by HIV-1 viral particles, may prove beneficial for gene therapy protocols. Overall gains in encapsidation and transduction efficiencies are clearly dependent upon a packaging system comprising both the Rev/RRE system and 5' UTR *cis *elements.

## Methods

### Plasmid Constructs

Murine leukemia virus (MLV) and MLV/HIV chimeric vector constructs were derived from the MLV vector, pLNCX [35]. The fundamental MLV vector as described in Figure 1A was developed as follows: i) the WPRE (woodchuck hepatitis virus posttranscriptional regulatory element) was first subcloned into the *EcoRI/HindIII *sites of a pCLNCX self-inactivating (SIN) vector [36]; ii) internal trans elements were replaced with a CMV-GFP cassette inserted by subcloning a *BamHI/XhoI *fragment from pTK113 (HIV-1 vector in Kafri lab), also retaining the HIV-1 cPPT-CTS (labeled cPPT in Figure 1A) in this fragment; iii) the 3' SIN LTR was replaced with a complete MoMLV 3' LTR from pLNCX by subcloning the *HindIII/PmeI *fragment; and iv) the firefly luciferase (originally obtained from pBI-GL, Clontech) was subcloned into the *BamHI *site of the MLV vector generating pTK1328, the basic MLV vector in Figure 1A. All notations of plasmids beginning with pTK are for ease of reference to the plasmid library in the Kafri lab.

All subsequent MLV/HIV chimeric constructs were derived from pTK1328 by inserting various HIV-1 *cis *elements. All HIV-1 *cis *elements were derived from a standard HIV-1 vector from our lab, such as pTK113 [37]. The MLV/HIV RRE vector (pTK1332) was derived from the basic MLV vector by subcloning a *BamHI *fragment (~850 bp) comprising the RRE into a *BamHI *site of pTK1328. The MLV/HIV RU5PS vector (pTK1442) was generated by subcloning a *BamHI *fragment into the *BamHI *site of pTK1328. The *BamHI *fragment was derived from a PCR amplified product originally cloned into pCR2.1-TOPO (Invitrogen) (pTK1428) and moved into the *SmaI/Acc65I *sites of pBlueScript (Strategene) (pTK1441). The PCR amplified product, comprising the HIV-1 R, U5, PBS, and canonical packaging signal which extends into the 5' end of Gag, was generated with the following primers: HIV RU5 For 5'-gcggccgcttaattaagggtctctctggttagaccagatctgagcc-3'; and HIV RU5Pack. Rev 5'-gcggccgcttgctgtgcgg-3'. The MLV/HIV RRE + RU5 vector (pTK1439) was constructed by subcloning a PCR amplified fragment into the *NotI *site of pTK1332. The PCR amplified product was formally cloned into pCR2.1-TOPO (Invitrogen) (pTK1427). The following primers were used to generate a PCR product comprising the HIV-1 R and U5 regions: HIV RU5 For primer as described above and HIV RU5 Rev 5'-gcggccgcactgctagagattttccacactgac-3'. The MLV/HIV RRE + PS vector (pTK1423) was constructed by subcloning a fragment, consisting of the canonical packaging signal and extending into the 5' end of Gag and HIV-1 RRE, into the *BamHI *sites of pTK1332. Upon cutting with *BamHI *the existing RRE in pTK1332 would not be retained. The last construct in the series, the MLV/HIV RRE + RU5PS vector (pTK1440), was generated by subcloning the *NotI *fragment from pTK1428 into the *NotI *site of pTK1332. The *NotI *fragment contains the HIV-1 R, U5, PBS, and canonical packaging signal extending into the 5' end of Gag.

The MLV/HIV RRE vector (pTK1086) described in Figure 7 was an earlier generation of pTK1332 above, which did not have the luciferase gene inserted. The packaging constructs supplying necessary structural/enzymatic proteins were 4XCTE Gag-Pol (kindly provided by the laboratory of Dr. Christopher Baum), or ΔNRF [37]. Expression of structural/enzymatic proteins from 4X CTE Gag-Pol is independent of all HIV-1 accessory genes, and encodes the complete *gag *and *pol *genes. The HIV-1 Rev protein was independently expressed from the EF-1α promoter in the E2F-Rev plasmid. The ΔNRF was described previously, but *gag-pol *gene expression is Rev-dependent. The envelope protein was supplied from pMD.G, a VSV-G expressing construct.

### Cells

293T cells were maintained in DMEM (Hyclone) supplemented with 10% FBS (Invitrogen). Media was also supplemented with a 100X Antibiotic-Antimycotic solution containing penicillin, streptomycin, and amphotercin B (Cellgro).

### Viral Particle Production and Concentration

Vector particles were produced by transient tranfection into 293T cells as described previously [38]. Briefly, each 10 cm dish of 293T cells was transfected with 15 μg vector, 10 μg packaging helper, 5 μg VSV-G envelope, and 5 μg Rev expressing plasmids. Plasmid amounts were compensated for in experiments in the absence of Rev with the empty plasmid construct, pCI-neo (Promega). Vector particles were harvested in conditioned media 48-60 hours post-transfection and filtered through a 0.45 μm filter. Vector titers were determined by serial dilution on 293T cells and scoring for GFP positive cells using a Leica Leitz DMIRB inverted fluorescent microscope. Vector titers are expressed as values normalized to p24 (HIV-1 viral particles), or reverse transcriptase activity (MLV viral particles). The p24 assay and the reverse transcriptase assays are described below. Concentration of vector particles was executed as done previously [38], with the exception that vector was purified over a single sucrose gradient, concentrated, and resuspended in 1X PBS.

### HIV-1 p24 Capsid Concentration

Details of this assay were described previously [39]. Briefly, EIA/RIA plates were coated with p24 antibody (NIH AIDS Research and Reference Reagent Program, #3537) at 1:1000 dilution and incubated overnight at 4°C. After blocking, samples/standards were treated with a 1% triton x-100 sample buffer, diluted appropriately, and added to plate for overnight incubation at 4°C. After washing, polyclonal rabbit anti-p24 antibody (NIH AIDS Research and Reference Reagent Program, #SP451T) at 1:300 was added to the plate, and incubated at 37°C for 3 hours. After washing, goat anti-rabbit IgG peroxidase (Pierce) at 1:15000 was added to the plate and incubated at 37°C for 2 hours. Assay was completed as described previously [39]. Data used for titer normalization, and determining amount of viral particles for RNA isolation, are a mean of replicate samples.

### MLV Reverse Transcriptase Assay

MLV reverse transcriptase activity was executed on vector particles (5 μl) harvested from the media by mixing with 25 μl of RT activity reagent (60 mM Tris pH8, 0.6 mM MnCl_2_, 90 mM KCl, 0.125 mg/ml Poly A [Roche], 6 μg/ml oligo dT_16_, 25 mM DTT, 0.06% Triton X-100, and 0.25 mCi/ml ^3^H-TTP [MP Biomedicals]). After incubation for 1 hour at 37°C the entire volume was spotted onto DE81 anion exchange chromatography paper (Whatman), and placed into 5% Na_2_PO_4 _for 5 minutes at room temperature. The samples were washed at room temperature five times, for 5 minutes each, with 5% Na_2_PO_4 _two times with water, and one time with 95% ethanol. The samples were dried, placed in scintillation fluid, and radioactivity was measured on a Beckman LS 6500 scintillation counter. Values were recorded as CPM. Data used for titer normalization, and determining amount of viral particles for RNA isolation, are a mean of replicate samples.

### FACS Analysis

Details of this assay were described previously [38]. Briefly, at the indicated times post-transduction cells were fixed, and GFP expression was assessed on a Dako CyAn flow cytometer at the University of North Carolina Flow Cytometry Core Facility. Data was analyzed with Summit v4.3.01 software (Dako).

### Luciferase Assays

Luciferase lysates were prepared by pelleting transfected 293T cells at the time of vector collection, and resuspending the pellet in 1X passive lysis solution (Promega). After freeze-thawing cell debris was pelleted by centrifugation for 15 minutes at 14,000 rpm and 4°C. Equivalent volumes of supernatant were assayed for firefly luciferase expression using 100 μl luciferin reagent (Promega). Luminescence was measured with a Victor^3 ^multilabel counter and Wallac 1420 Workstation software (Perkin-Elmer). Results are expressed as relative light units (RLU)/mg protein. Protein concentrations were assayed according to manufacturer's instructions for Pierce BCA Protein Assay kit.

### RNA Isolation

At 48-60 hours post-transfection vectors were harvested from the media, and cells were divided for luciferase assay, total protein, and cytoplasmic RNA/protein fractionation. Cells were removed from the plate by trypsinizing, and pelleted. The cytoplasmic fraction was separated by treating the pelleted cells with a MES (2-(*N*-morpholino)ethanesulfonic acid) buffered 0.1% Triton X-100 solution (10 mM MES pH 6.5, 60 mM KCl, 15 mM NaCl, 5 mM MgCl_2_, 250 mM sucrose, 0.1% Triton X-100) + protease inhibitor minicocktail (Roche) + 200 units/ml RNase Inhibitor (Fermentas). Pellets were incubated 2 minutes on ice, and nuclei were pelleted at 1500 rpm and 4°C. The cytoplasmic supernatant fraction was collected and separated for RNA isolation and protein analysis. Cytoplasmic fraction was monitored by western blot analysis for the absence of nucleolin protein, compared to total cellular proteins. RNA was purified from the cytoplasmic supernatant using the PARIS protein and RNA isolation kit (Ambion). Manufacturer's instructions were followed starting with addition of the 2X lysis/binding solution to an equal volume of cytoplasmic supernatant. Purified RNA was treated with DNase I Turbo (Ambion) for 1.5 hours at 37°C, and inactivated according to manufacturer's instructions. RNA integrity and concentration were regularly assessed by agarose gel electrophoresis. Cytoplasmic RNA was then utilized for analysis in qRT-PCR and northern blot.

Isolation of RNA from vector particles was carefully executed so that all vectors packaged into HIV-1 viral particles were normalized for equivalent amounts of p24 prior to isolation. For each sample at the time of RNA isolation p24 equivalents of vector particles and 5 × 10^6 ^293T cells were concomitantly added to the RLT lysis solution in preparation for column purification of the RNA with the RNeasy Plus Mini kit (Qiagen). Spiking the vector particles with 293T cells at time of purification has multiple advantages: i) 293T cell RNA is a carrier during RNA purification; ii) RNA can be easily quantified after purification; and iii) quantity and integrity of purified RNA can be monitored by agarose gel electrophoresis. For purposes of homogenization, the lysed particles/293T cell RNA were passed through a QIAshredder column (Qiagen) and subsequently through a genomic DNA eliminator column provided with the RNeasy plus mini kit. Lysate was then purified through the RNA isolation column provided with the RNeasy plus kit. Purified RNA was treated with DNase I Turbo (Ambion) as described for cytoplasmic RNA. Vector particle RNA was then utilized for analysis in qRT-PCR and northern blot. RNA was isolated from MLV viral particles in a similar fashion, except that viral particles were normalized for equivalent amounts of MLV reverse transcriptase. The reverse transcriptase assay is described above.

### qRT-PCR

Purified cytoplasmic and vector RNA were heated to 65°C for 5 minutes prior to preparing each reverse transcription (RT) reaction. Each RT reaction was prepared at final concentrations of 1X RT Buffer (Qiagen OmniScript kit), 0.5 mM each dNTP, 10 μM random prime nonamer, 10 units RNase Inhibitor (Fermentas), 500 ng RNA, and 4 units OmniScript reverse transcriptase (Qiagen). As controls for the presence of contaminating DNA each reaction was also performed in the absence of reverse transcriptase. Reactions were executed for 1 cycle on a BioRad MyCycler at 37°C for 1 hour. Equivalent amounts of cDNA were used in the subsequent quantitative PCR reaction. Each qPCR reaction was prepared at final concentrations of 1X ABI Taqman mix (2X Taqman Gene Expression Master Mix, Applied Biosystems), 0.9 μM forward primer, 0.9 μM reverse primer, and 0.1 μM probe. Reactions were performed at 1 cycle of 50°C/2 minutes, 1 cycle of 95°C/10 minutes, and 40 cycles of 95°C/15 seconds + 60°C/30 seconds on a 7300 real time PCR system (Applied Biosystems). Vector RNAs were detected with a primer/probe set to the luciferase gene: For Luc 5'-aggtcttcccgacgatga-3', Rev Luc 5'-gtctttccgtgctccaaaac-3', and probe #70 (Roche Universal Probe Library). All quantitative PCR reactions were normalized to an endogenous control reaction for TATA Binding Protein (TBP) using the following primer/probe set in independent reactions: For TBP 5'-gaaccacggcactgattttc-3', Rev TBP 5'-tgccagtctggactgttcttc-3', and probe #92 (Roche Universal Probe Library).

The levels of vector RNA (VRNA) and cytoplasmic RNA (CRNA) derived from the qRT-PCR data are expressed as arbitrary units (AU). The values were determined by normalizing the cycle threshold (C_t_) for luciferase to that obtained for TBP, from each reaction. The calculation was as follows: 2^-ΔCt^, where the ΔC_t _is (Luc C_t _- TBP C_t_). Independent reactions were performed for each VRNA and CRNA sample. All isolated VRNA could be normalized to TBP since each sample was copurified with 293T cell RNA, as described above. Calculation of the encapsidation efficiency, where VRNA relative to CRNA is expressed as AU, was determined for each independent sample. The calculation was as follow: 2^-ΔΔCt^, where the ΔΔC_t _is (VRNA Luc C_t _- VRNA TBP C_t_) - (CRNA Luc C_t _- CRNA TBP C_t_). All relative encapsidation efficiencies are expressed as an average of at least three independent experiments with corresponding standard deviations (S.D.).

### Northern Blot Analysis

Cytoplasmic RNA was isolated as described, and vector RNA was isolated from concentrated vector particles. Vector RNA was prepared from equivalent levels of p24, and at the time of isolation the samples were copurified with 293T cells as described above. Prior to resolving RNA on a denaturing formaldehyde agarose gel, RNA was denatured at 65°C for 10 minutes. Equivalent amounts of cytoplasmic RNA, and vector RNAs, were resolved on the gel. Northern blot analysis was performed under standard conditions. Probes were random prime labeled with α^32^P-dCTP (Easy Tide Deoxycytidine 5'-triphosphate, Perkin-Elmer) at 37°C for 1 hour. Probes were either a BstEII fragment from pTK1440 generated to the 5' end of the vector RNA, or to the GFP gene located in the 3' end of the RNA. Images were obtained on BioMax MR film (Kodak), or by phosphorimager (Molecular Dynamics Storm System).

### Western Blot Analysis

Cytoplasmic and total cell lysates were resolved by standard denaturing SDS-PAGE analysis on 10% gels and blotted to Hybond P membrane. Membranes were probed with rabbit anti-nucleolin (1 ug/ml; Abcam, cat# ab22758-100) and rabbit anti-GAPDH (1:500; Santa Cruz Biotechnology, cat# FL-335). Detection was achieved by probing blots with goat anti-rabbit IgG peroxidase (1:40000; Pierce). All antibody incubations and washes were done in 1X PBST. Signals were detected using ECL (GE Healthcare).

### Southern Blot Analysis

293T cells were transduced with MLV/HIV RRE + RU5PS vector packaged into HIV-1 or MLV viral particles, at a MOI of 5. Total cellular DNA was isolated at 5 days post-transduction (referred to as P0, or no cell passages, in figures), and after five passages (P5) of the transduced cells. To collect total DNA transduced cells were lysed in a proteinase K solution (10 mM Tris pH 8, 10 mM EDTA pH 8, 0.5% SDS, 0.4 M NaCl, and 200 μg/ml proteinase K) for 48 hours at 55°C. Total DNA was extracted with v/v phenol (Invitrogen) and v/v phenol/chloroform/isoamyl alcohol (Invitrogen) and then treated with RNase A (20 μg; Fermentas) at 37°C for 2 hours. A second round of extractions was performed and total DNA was precipitated using standard ethanol precipitation.

Isolated gDNA (10 μg) was digested with BsrGI and DpnI for ~24 hours at 37°C. DpnI was included to eliminate putative plasmid DNA carry-over from the transfections during vector production in 293T cells. Equivalent amounts of gDNA were resolved on 1% agarose gels, transferred to Zetaprobe membrane, and probed with a BstEII fragment from pTK1440 that recognizes the 5' end of the vector enabling a size distinction between linear, 1-LTR, 2-LTR and backbone/integrated forms. Images were captured on BioMax MR Film (Kodak), or by phosphorimager (Molecular Dynamics Storm System).

### Real Time qPCR

Total cellular DNA was isolated as described for Southern blot analysis and qPCR for vector and β-globin were described previously [39]. Briefly, vector copy number was derived from standard curve produced from FLP9 cells, which contain a single copy of HIV-1 vector per diploid genome. One nanogram of total cellular DNA was previously calculated to contain 303 copies for β-globin per diploid genome, or 151.5 copies vector per diploid genome. Primers for vector copy number were designed to the WPRE region, which is also in our MLV/HIV chimeric vectors. All primers and PCR reaction conditions were described previously [39].

### Mouse Subjects

Female C57Bl/6 mice (n = 10), 3 months old (Jackson Labs) were housed in standard conditions with 3-4 mice per cage with food and water available *ad libitum*. All procedures were in accordance with the Guide for the Care and Use of Laboratory Animals (DHHS Publication No. (National Institutes of Health) 85-23), and all procedures received prior approval by the NIH Institutional Animal Care and Usage Committee. All procedures were performed according to animal protocol number, 396-LNS-2014.

### Stereotaxic Administration of Retroviral Vectors

Mice were anesthetized (Avertin 0.02 mg/ml, 0.5-1.0 ml injection per mouse), and MLV retrovirus (n = 5 mice), or HIV lentivirus (n = 5 mice) was injected stereotactically (2 μl using a 5 μl Hamilton microliter syringe) into the right striatum (AP = 1.0 mm anterior from bregma; lateral = 1.5 mm; ventral = 3.0 mm). Studies were executed with the MLV/HIV RRE vector (Figure 7A) packaged into MLV viral particles, or HIV-1 viral particles generated from the ΔNRF [37] helper construct. One month thereafter animals were given an overdose of anesthetics and perfused transcardially with cold 4% paraformaldehyde (PFA) in 0.1 M phosphate buffered saline (PBS). After 24 hr, brain tissue was equilibrated in 30% sucrose. Sequential horizontal sections (40 μm) using a sliding freezing microtome (HM450, ThermoFisher) were taken through the extent of the striatum and stored in phosphate buffered glycerol at -20°C.

### Immunohistochemistry and Confocal Microscopy

Double labeling for the neuronal marker NeuN and GFP was done on a 1:6 series of 40-μm free-floating horizontal sections as described previously [40]. Sections were washed and blocked in TBS with 3% donkey serum and 0.3% Triton X-100 (TBS-plus). Primary antibodies raised in two different species were pooled in TBS-plus and incubated for 48 h at 4°C. The neuronal marker NeuN [mouse monoclonal antibody, (Millipore) 1:100] was combined with antibody for GFP [rabbit polyclonal antibody (Millipore), 1:1000]. Corresponding secondary antibodies [donkey anti-mouse CY3 and goat anti-rabbit Alexa488 (Jackson ImmunoResearch Inc.), 1:250] were pooled, and sections were incubated for 4 h at room temperature following washing in TBS-plus. Sections were mounted and coverslipped with DABCO-PVA. Sections were imaged by confocal scanning laser microscopy (MPE1000, Olympus and Laser Scanning Zeiss 510 Meta). Brain sections with GFP positive cells were captured with the Zeiss LSM Image Browser software and used to score for GFP, NeuN, and colocalization of the two markers by scanning through the z-axis. Scoring represents positive cells from brain sections of mice transduced with the MLV/HIV RRE vector packaged into MLV (n = 5), or HIV-1 (n = 5), derived viral particles.

## Competing interests

The authors declare that they have no competing interests.

## Authors' contributions

ASC, HvP and TK designed research, analyzed data and wrote the paper; ASC, HvP and NS performed research; HM contributed reagents and necessary materials. All authors read and approved the final manuscript.

## Supplementary Material

Additional file 1 **Figure S1. Cytoplasmic separation and transfection assessment**. **A**. At the time of harvesting vectors total and cytoplasmic protein fractions were routinely collected from the producer 293T cells to monitor separation of the cytoplasmic fraction. Equivalent amounts of total (TP) and cytoplasmic (CP) protein lysates were analyzed for nucleolin (a nuclear specific protein), and GAPDH, by western blot analysis. Three independent experiments are shown for the MLV/HIV RRE + RU5PS vector packaged into HIV-1 particles in the presence of Rev. **B**. Cytoplasmic RNAs isolated from transfected producer 293T cells at the time of vector harvest. Four independent experiments (lanes 1-4) were analyzed by denaturing northern blot analysis. The MLV/HIV RRE + RU5PS vector RNA is shown in the absence of Rev (two separate experiments; lanes 1 and 2) and in the presence of Rev (two separate experiments; lanes 3 and 4). Dilutions of vector RNA in lane 4 were also resolved at 50% (lane 5), and 20% (lane 6). Actin was probed as a positive control. The band densities were quantitated by phosphorimager for vector **(C) **and actin **(D)**. Numbers below each bar correlate with lanes on northern blots. Click here for file
