# Supplementary figures and images for: The HIV-1 Rev/RRE system is required for HIV-1 5' UTR cis elements to augment encapsidation of heterologous RNA into HIV-1 viral particles
Source: Retrovirology. 2011 Jun 24;8:51. doi: 10.1186/1742-4690-8-51 (PMC3131246; doi:10.1186/1742-4690-8-51)

## Slide 1
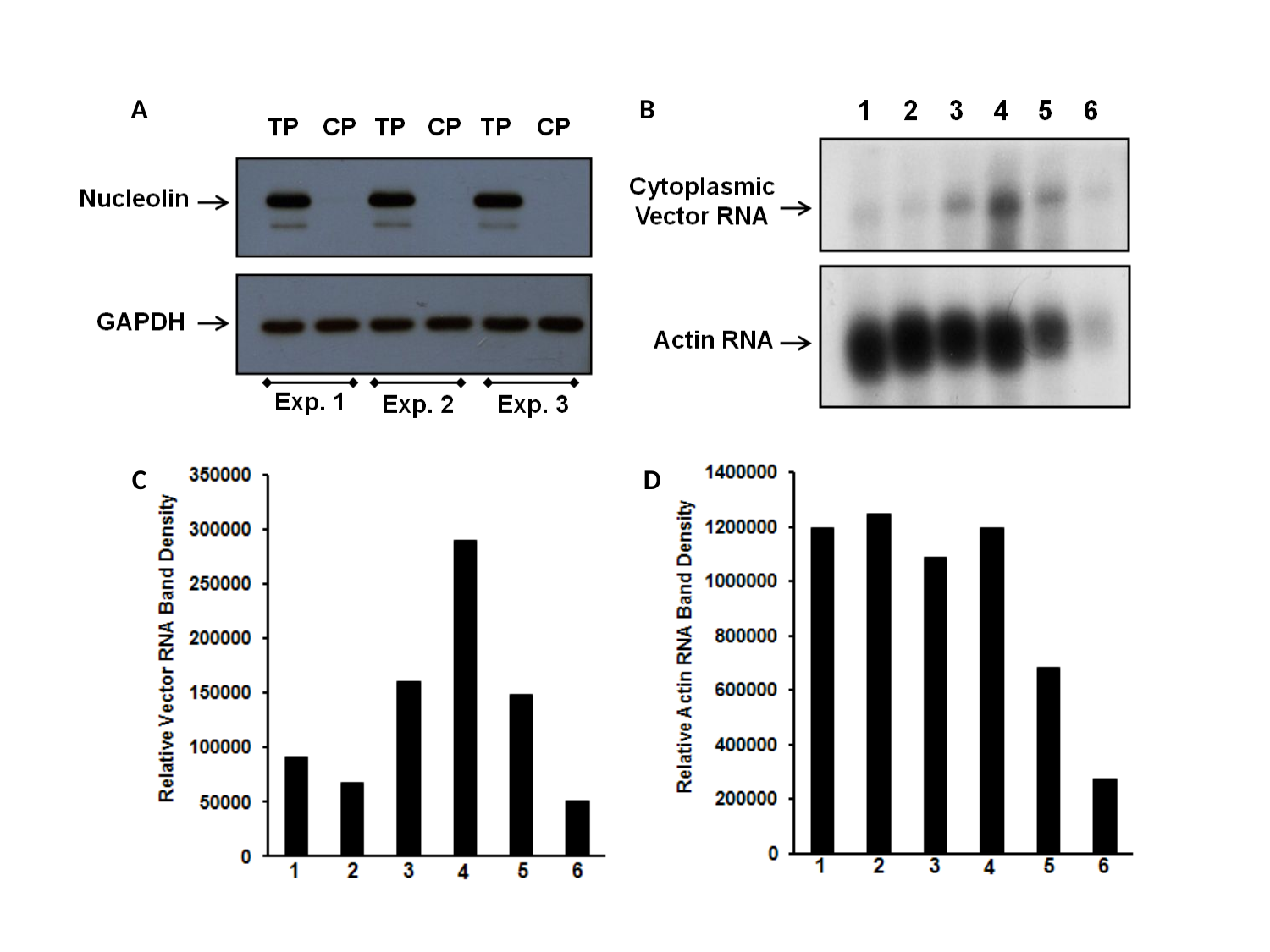

A
B
C
D

Supplement: Additional file 1 — Figure S1. Cytoplasmic separation and transfection assessment. A. At the time of harvesting vectors total and cytoplasmic protein fractions were routinely collected from the producer 293T cells to monitor separation of the cytoplasmic fraction. Equivalent amounts of total (TP) and cytoplasmic (CP) protein lysates were analyzed for nucleolin (a nuclear specific protein), and GAPDH, by western blot analysis. Three independent experiments are shown for the MLV/HIV RRE + RU5PS vector packaged into HIV-1 particles in the presence of Rev. B. Cytoplasmic RNAs isolated from transfected producer 293T cells at the time of vector harvest. Four independent experiments (lanes 1-4) were analyzed by denaturing northern blot analysis. The MLV/HIV RRE + RU5PS vector RNA is shown in the absence of Rev (two separate experiments; lanes 1 and 2) and in the presence of Rev (two separate experiments; lanes 3 and 4). Dilutions of vector RNA in lane 4 were also resolved at 50% (lane 5), and 20% (lane 6). Actin was probed as a positive control. The band densities were quantitated by phosphorimager for vector (C) and actin (D). Numbers below each bar correlate with lanes on northern blots. [file 1742-4690-8-51-S1.PPT]
